# Changes in Segmental Impedances and Selected Body Composition Parameters Assessed by Multi-Frequency Bioimpedance Analysis after Fluid Consumption in Healthy Young Population

**DOI:** 10.7150/ijms.77396

**Published:** 2023-11-07

**Authors:** Martin Forejt, Kateřina Pokorová, Michal Uher, Jan Novák, Erika Čermáková

**Affiliations:** 1 Department of Public Health, Faculty of Medicine, Masaryk University, Kamenice 5, 625 00 Brno, Czech Republic.; 2 Institute of Mathematics and Descriptive Geometry, Faculty of Civil Engineering, Brno University of Technology, Žižkova 17, 602 00 Brno, Czech Republic.; 3 Research Centre for Applied Molecular Oncology (RECAMO), Masaryk Memorial Cancer Institute, Zluty kopec 7, 656 53 Brno, Czech Republic.; 4 Second Department of Internal Medicine, Faculty of Medicine, St. Anne's University Hospital and Masaryk University, Pekařská 664/53, 65691 Brno, Czech Republic.; 5 Department of Physiology, Faculty of Medicine, Masaryk University, Kamenice 5,625 00 Brno, Czech Republic.

**Keywords:** body composition changes, segmental multi-frequency bioelectrical impedance analysis, acute fluid consumption, percentage of body fat, visceral fat, adults

## Abstract

**Objectives:** Body composition (BC) analysis is a routine part of comprehensive public health care. Assessment of BC is more important source of information than BMI. Adherence to the standard measurement conditions is essential for the correct results. Our study aimed to examine the effect of acute fluid consumption on measures of body mass (BM), percentage of body fat (%BF), visceral fat (VF), percentage of body water (%BW), and impedance at 100 kHz (I100) and 20 kHz (I20) using segmental multi-frequency bioelectrical impedance analysis (SMF-BIA) in a general healthy population.

**Methods:** 95 consecutive healthy normal-weight adults (42 men; 53 women) were involved in the study (mean ± s.d.; age 23.9±1.6 years; body mass 68.3±14.1 kg). All subjects underwent two separate series of body composition (BC) measurements at 0 (BASELINE), 30, 60, 90 min (POST): the first series after drinking 600 ml of isotonic carbohydrate/electrolyte drink (IST) and the second after no fluid administration (CON). Individual measurements were performed in the morning on two consecutive days.

**Results:** In the IST group, BM, VF (both P<0.001), and %BF (P<0.05) increased significantly at 30 min POST compared to BASELINE. BM and VF remained elevated at 90 min POST (both P<0.001). %BW decreased significantly at 30 min POST (P<0.01) then increased at 60 min (P<0.001) and 90 min (P<0.01) POST. There were no significant changes in I100. I20 increased significantly at 30 min POST (P<0.001) then decreased at 60 min (P<0.001) and 90 min POST (P<0.01) compared to BASELINE. In the CON group, BM and VF decreased below BASELINE at 90 min POST (P<0.001), %BF, %BW and I100 did not change significantly. The difference between IST and CON was statistically significant for all POST measurement times only in BM and VF (both P<0.001). The VF results are also underlined by the detected impedance changes in the trunk area at 20 kHz (B20) and 100 kHz (B100) at 60 min and 90 min (both P<0.001)**.**

**Conclusions:** Our study suggests that segmental impedances and BC measurement in healthy young normal-weight adults requires strict adherence to fluid restriction at least 90 min before the measurement to avoid false impedance values and overestimation of BM and VF.

## Introduction

Body composition (BC), mainly percentage of body fat (%BF) and visceral fat (VF), are important indicators of the health and nutritional status in the public health care (i.e., in preventive measures to determine overweight, obesity, and concerning other diseases in whose BC knowledge is beneficial) 1. Traditionally used parameter body mass index (BMI), does not reflect BC precisely - while in muscular men, it may be falsely overestimated, even though the %BF is normal; in women, with a lower proportion of muscle mass, it may be falsely underestimated, even though the %BF would already reflect overweight 2,3. For these reasons, the calculation of traditional BMI is used mainly as a public screening method. At the same time, in scientific research and daily clinical practice, bioelectric impedance analysis (BIA) is more often used for the complex evaluation of BC. BIA devices use alternating electric current, and individual tissues show different resistance to it 1. If properly used, BIA provides reliable and reproducible information about BC 4. In the last years, the segmental multi-frequency BIA (SMF-BIA) has started to be used more frequently. Compared to the original BIA, SMF-BIA measures individual body segments using multiple current frequencies and thus provides even more detailed information about BC in individual body parts. SMF-BIA is easy, fast, cheap 1,4,5, and accurate if we follow standardized measurement conditions 6,7. One of the main recommendations is not to drink for 4 hours before the measurements 8 as drinking can reduce the reliability of results. This recommendation is based on numerous studies examining the traditional BIA that have reported significant changes in total body water (BW) 9,10,11,12,13,14, %BF 9,10,11,12,15,16 and impedance 9,13,14,15 after fluid intake. However, these studies differed in the quantity and composition of the beverage used, the length of the follow-up period, and the BIAs´ method used. Therefore, it is necessary to review and specify the conditions of SMF-BIA measurements after fluid consumption.

The main goal of this study was to observe the changes in segmental impedances and selected body composition parameters when choosing a longer (90 minute) time interval between isotonic carbohydrate/electrolyte drink (IST) intake and BC analysis than it was in the studies carried out so far, on a larger set of participants than described by most studies listed in the publication and while maintaining the same measurement technique (BIA) and a similar amount of drink.

## Subjects and Methods

### Subjects

A total of 95 healthy young adults (42 men, 53 women), aged 23.9 ± 1.6 years (mean ± s.d.) voluntarily participated in the research. The study was approved by the Ethics Committee of the Faculty of Medicine of Masaryk University. Participants signed an informed consent form before the study. Obtained data were anonymized before processing. The study was performed following the Declaration of Helsinki.

### Study procedures

Each participant was invited to the examination room for SMF-BIA measurement on two consecutive days. The measurements were performed according to the traditional BIA and manufacturer protocol. Participants were informed in advance of the measurement conditions and had to follow several fundamental standard guidelines 8: no food, drink (4 h before), no exercise, sports (12 h before), no alcohol (2 days before), no diuretics (7 days before). During the measurements, they sat quietly in the examination room at a controlled temperature of 22 °C, and except for study-related fluids, they were not allowed to drink and eat anything. They should be emptied 30 min before measurement. Further emptying of the bladder/gut was allowed after the last measurement. On the first day (IST), participants drank 600 ml of Isostar Energy Sports Drink within 5 minutes; on the second day (CON), they drank nothing. The drink was selected because of its high energy and electrolyte content. 100 g of isotonic instant powder dissolved in water contained 384 kcal, 95 g carbohydrates, 190 mg sodium, vitamin E 12 mg, C 76 mg, and B1 0.54 mg, osmolality 290 mOsm/kg, temperature 5 °C. Cold temperature was selected as it increases gastric emptying rate 10.

### Anthropometric measurements

We performed body height measurement (SECA 764 - SECA GMBH & CO., Hamburg, Germany) and SMF-BIA (InBody 230 and software Lookin´Body 3.0 - Biospace, Seoul, Korea) to determine the anthropometric characteristics of the participants. Trained specialists carried out the examinations. Participants wore only tight-fitting swimwear. Body height was measured on the first morning. Participants were then asked to sit on a chair with their legs resting comfortably on the floor 5 min before the measurements to equilibrate body fluids. During the measurements, the participants stood erect holding the handgrips with electrodes, and their bare feet were correctly placed on the contact electrodes of the SMF-BIA device. The procedure for BC measuring was as follows: after an initial body composition measurement at 0 min (BASELINE), the subjects consumed 600 ml of drink or nothing, and they were then measured at 30 (30ˈ), 60 (60ˈ) and 90 min (90ˈ) after BASELINE (POST).

### Statistical methods

The data were analysed using the R language (R: A language and environment for statistical computing, R Core Team, R Foundation for Statistical Computing, Vienna, Austria). All values are expressed as mean ± s.d. unless otherwise noted. We used the Shapiro-Wilk normality test for normality testing and Bartlett's test for homoscedasticity testing. The variance of the differences between groups was tested using Mauchly's test of sphericity. If the sphericity assumption was not assumed, the Greenhouse-Geisser sphericity correction was applied. Statistical significance was established at P<0.05 for all the analyses. The dependence of BM, %BF, VF, %BW, the whole (I100, I20) and segmental body impedance (RA100, LA100, B100, RL100, LL100 and RA20, LA20, B20, RL20, LL20) on measurement time and consumed fluid was analysed using a two-factor repeated-measures analysis of variance. These factors included measurement time with four levels (BASELINE, 30ˈ 60ˈ, 90ˈ) and ingested fluids with two levels (IST and CON).

Pairwise comparisons using Bonferroni correction were performed to determine the between means in case of significant results of analysis of variance.

If data did not meet the normality assumption, the analysis was made on transformed data. Appropriate transformations were chosen by considering the type of violation of symmetry or normality in order to satisfy the assumption. As the data for **BM** and **VF** do not meet the normality assumption due to asymmetry, the transformations 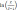 and 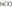 were performed, respectively. In the case of data for RA20, RA100, LA20 and LA100 that do not meet the normality assumption due to excess kurtosis, the transformations 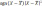 with 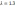 was performed.

The data analysis with and without the assumption of normality gave very similar p-values; therefore, the results are presented as non-transformed data.

## Results

Characteristics of observed variables show Table 1.

We focused on the dependence of the observed variables on measurement times in IST and CON. Firstly, we will always describe the interaction of consumed fluid and time factor; for the statistically significant result, we will describe the differences between IST and CON. Secondly, we will comment on the time effect for each observed variable in IST and CON.

**Figure 1:**
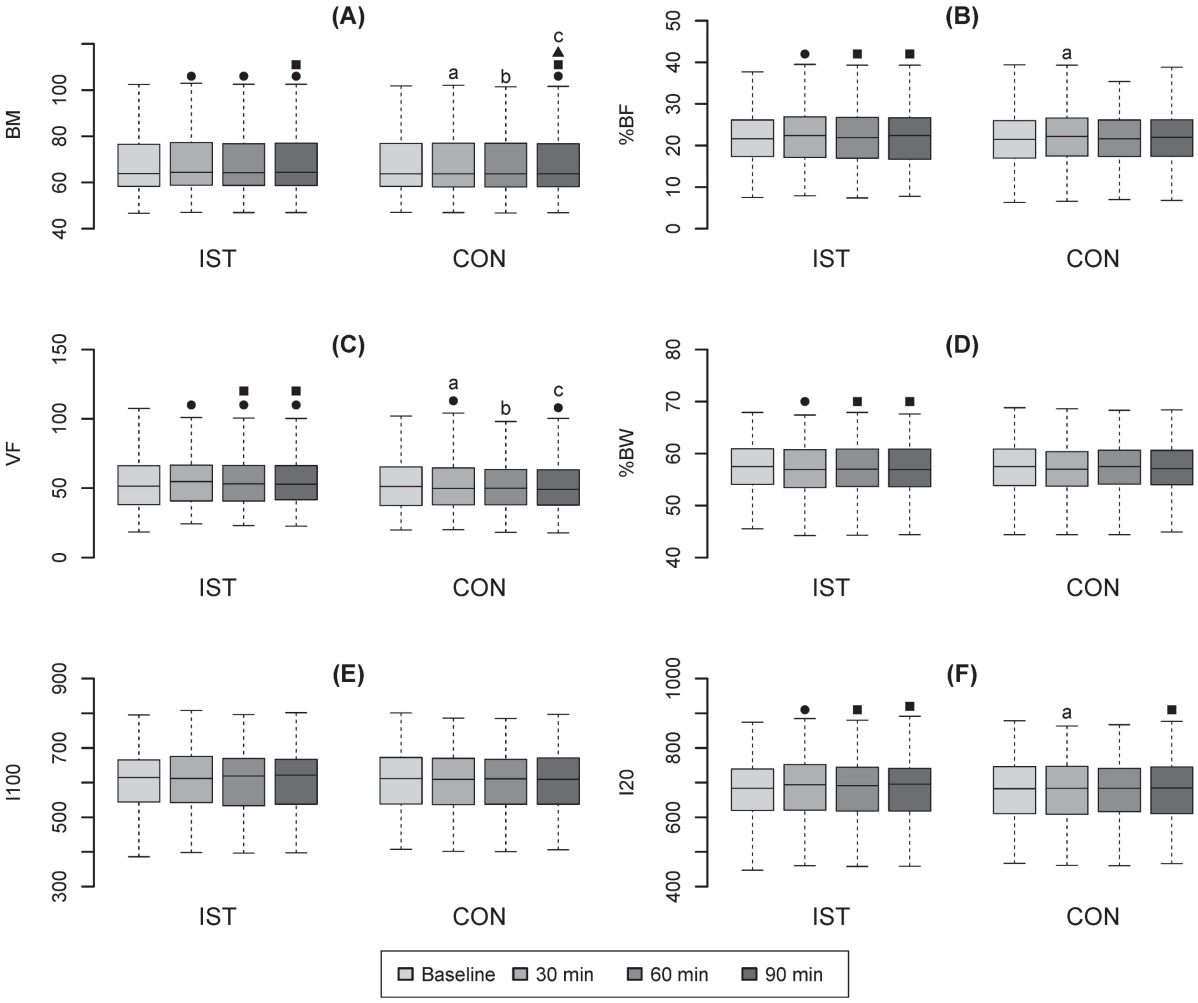
Boxplots of values over time in IST and CON (mean ± s.e.m.) for (A) BM, (B) %BF, (C) VF, (D) %BW, (E) I100, (F) I20. ● Significantly different from BASELINE, ■ significantly different from 30ˈ, ▲ significantly different from 60ˈ. Also, **a**, significantly different from 30ˈ IST, **b**, significantly different from 60ˈ IST, **c**, significantly different from 90ˈ IST.

The interaction of consumed fluid and time factor for BM was statistically significant (P<0.001) (Figure 1A). The post-hoc analysis indicated the statistically significant difference in BM (P<0.001) between IST and CON measurements at 30ˈ POST (IST: 68.7±14.1; CON: 68.2±14.3), 60ˈ POST (IST: 68.6±14.2; CON: 68.2±14.2) and 90ˈ POST (IST: 68.6±14.1; CON:68.1±14.2).

The time effect was also statistically significant (P<0.001). BM remained elevated over the baseline value (68.2±14.2) at 30ˈ, 60ˈ, 90ˈ POST in IST (all P<0.001). On the other hand, a slight decrease of BM between 30ˈ and 90ˈ POST appeared in IST (P<0.05). In CON, the BM decreased under the baseline value (68.2±14.3) at 90ˈ POST (P<0.001), further, it decreased under the value of 30ˈ and 60ˈ at 90ˈ POST (both P<0.01).

The interaction of consumed fluid and time factor for **%BF** was statistically significant (P<0.001) (Figure 1B). The post-hoc analysis indicated that there was a statistically significant difference in %BF between IST (22.4±6.9) and CON (21.9±6.8) only at 30ˈ POST (P<0.05). The time effect was also statistically significant (P<0.001). In IST, the %BF increased over the baseline value (21.9±6.8) at 30ˈ (P<0.001), further, it decreased under the value of 30ˈ at 60ˈ (22.0±6.8; P<0.01) and 90ˈ (22.0±6.9; P<0.001) POST.

The interaction of consumed fluid and time factor for **VF** was statistically significant (P<0.001) (Figure 1C). The difference between IST and CON was statistically significant (P<0.001) for all POST measurement times (IST at 30ˈ: 58.1±25.8; CON at 30ˈ: 54.7±26.3; IST at 60ˈ: 57.2±25.9; CON at 60ˈ: 54.8±26.1; IST at 90ˈ: 57.1±25.8; CON at 90ˈ: 54.6±25.8).

The time effect was also statistically significant (P<0.001). In IST, the post-hoc analysis indicated a statistically significant increase of VF over the baseline value (55.4±25.9) at 30ˈ POST (P<0.001), then it decreased but still stayed over the baseline value to the end of observation (both P<0.001). A statistically significant decrease under the value at 30ˈ POST was indicated in 60ˈand 90ˈ POST (both P<0.001). In CON, a statistically significant decrease under the baseline value (55.3±26.5) was detected at 30ˈand 90ˈ POST (both P<0.05).

The interaction of consumed fluid and time factor for **%BW** was not statistically significant (Figure 1D); there were also no statistically significant differences between IST and CON. The time effect was statistically significant (P<0.01). In IST, the post-hoc analysis indicated a statistically significant drop of %BW under the baseline value (57.3±5.0) at 30ˈ (56.8±5.0) POST (P<0.01). Then, the values increased above the value obtained at 30ˈat and 60ˈ (57.1±5.0) POST (P<0.001) and at 90ˈ (57.1±5.0) POST (P<0.01).

The interaction between consumed fluid and time factor for **I100** was not statistically significant (P=0.117), the main time factor was not statistically significant either (P=0.216). The values for I100 are presented in Figure 1E. The interaction between consumed fluid and the time factor for **I20** was statistically significant (P<0.001) (Figure 1F). Statistically significant (P<0.001) differences between IST and CON appeared at 30ˈ (IST: 684.3±93.7; CON: 676.2±90.5). The time effect was also statistically significant (P<0.01). The post-hoc analysis revealed statistically significant (P<0.001) increase of I20 at 30ˈ POST over the baseline value (678.9±90.1), then its decrease under the value of 30ˈ appeared at 60ˈ (681.4±92.2; P<0.001) and at 90ˈ (681.4±90.6; P<0.01) in IST. In CON, the only statistically significant decrease under the value of 30ˈ was detected at 90ˈ POST (679.1±90.4; P<0.01).

We also focused on segmental impedance values (Table 1) and did the same analysis.

First the results for segmental impedance values in trunk.

The interaction of consumed fluid and time factor for **B100** was statistically significant (P<0.001). The difference between IST and CON was statistically significant (P<0.01 at 30ˈ, P<0.001 at 60ˈ and 90ˈ) for all POST measurement times (IST at 30ˈ: 22.0±2.8; CON at 30ˈ: 22.3±2.8; IST at 60ˈ: 21.9±2.7; CON at 60ˈ: 22.5±2.8; IST at 90ˈ: 21.9±2.7; CON at 90ˈ: 22.4±2.8).

The time effect was also statistically significant (P<0.001). In IST, the post-hoc analysis indicated a statistically significant drop of B100 under the baseline value (22.4±2.9) at 30ˈ, 60ˈ and even at 90ˈ POST (all P<0.001). In CON, there was a statistically significant increase of B100 over the baseline value (22.3±2.8) at 60ˈ and at 90ˈ POST (both P<0.05).

The results for B20 are very close to those of B100.

The interaction of consumed fluid and time factor for **B20** was statistically significant (P<0.001). The difference between IST and CON was statistically significant (P<0.01 at 30ˈ, P<0.001 at 60ˈ and 90ˈ) for all POST measurement times (IST at 30ˈ: 25.7±2.9; CON at 30ˈ: 26.1±2.8; IST at 60ˈ: 25.5±2.7; CON at 60ˈ: 26.2±2.8; IST at 90ˈ: 25.6±2.7; CON at 90ˈ: 26.3±2.9).

The time effect was also statistically significant (P<0.001). In IST, the post-hoc analysis indicated a statistically significant decrease of B20 under the baseline value (26.2±3.0) at 30ˈ, 60ˈ and even 90ˈ POST (all P<0.001). In CON, there was a statistically significant increase of B20 over the baseline value (26.0±2.9) at 60ˈ and at 90ˈ POST (both P<0.01).

The results for segmental impedance values in legs.

The interaction of consumed fluid and time factor for **RL20** and for **RL100** was statistically significant (P<0.05 for RL20, P<0.01 for RL100). The difference between IST and CON was statistically significant only for combinations of POST measured times in IST and CON we are not interested. The time effect was not statistically significant neither for RL20 nor RL100.

In **LL20** and **LL100**, no statistically significant interaction of consumed fluid and time was found. The time effect was not statistically significant in LL20 and in LL100 too.

The results for segmental impedance values in arms.

The interaction of consumed fluid and time factor for **RA20** was statistically significant (P<0.001). The difference between IST and CON was statistically significant only at 30ˈ POST (P<0.001; IST: 376.0±63.1; CON: 369.7±60.5).

The time effect was also statistically significant (P<0.001). In IST, the post-hoc analysis indicated a statistically significant increase of RA20 over the baseline value (370.0±59.4) at 30ˈ and 60ˈ POST (P<0.001 at 30ˈ, P<0.05 at 60ˈ). In CON, there was a statistically significant increase of RA20 over the baseline value (368.9±58.9) only at 90ˈ POST (P<0.05).

The interaction of consumed fluid and time factor for **LA20** was statistically significant (P<0.001). The difference between IST and CON was statistically significant only at 30ˈ POST (P<0.01; IST: 382.6±64.2; CON: 376.5±61.6).

The time effect was also statistically significant (P<0.001). In IST, the post-hoc analysis indicated a statistically significant increase of LA20 over the baseline value (377.7±60.5) only at 30ˈ POST (P<0.001). In CON, there was no statistically significant time effect.

In **RA100** and **LA100**, no statistically significant interaction of consumed fluid and time was found. The time effect was not statistically significant in RA100 and in LA100 too.

## Discussion

In the current study we examined how the results of BC measurement with SMF-BIA are affected by previous consumption of 600 ml of IST at several time points in healthy young adults. In general, it is recommended by the manufacturer not to eat or drink for 4 hours before measuring with SMF-BIA to obtain accurate results 8. However, we wanted to determine whether this interval is not inadequately long and whether it is possible to get reliable results of the SMF-BIA BC analysis even after a shorter period of time. This issue has already been addressed in several studies, which will be discussed further.

Our study population consisted of young, healthy adults to minimize possible distortion of BC parameters by various diseases or water imbalance (e.g., in patients with swellings), as pointed out in several studies 6,8,18,19. The number of participants in our study was higher than in many previously published studies 9,10,11,13,15,16,17, and we followed them at multiple time intervals similarly to some studies 16,17 but for a longer time after IST drinking compared to most of the previous studies 9,10,16,17. Interval of more than 90ˈ was not possible as we found that a number of study participants were no longer able to hold urine and needed to look for the toilet, which was certainly related to the previous intake of 600ml of fluids. Unfortunately, this would lead to a methodical error. A possible question for reflection is to change the design of the study by reducing the amount of fluids consumed, which could lead to the possibility of extending the time interval.

The amount of IST used was almost similar to amount used in other studies 9,10,13,17. Such a study design enabled us to obtain a more extensive set of data with the broadest available timeline to study changes of IST drinking on the studied BC parameters.

In our study, we found a statistically significant difference in **BM** between IST and CON (P<0.001). BM remained elevated after the IST drinking throughout the observation period (P<0.001). In CON, the BM decreased under baseline value at 90ˈ POST (P<0.001), which can be attributable to the ongoing catabolic processes in the human body.

The most frequently investigated parameter in studies with a similar design was **%BF**. In IST, we found a statistically significant increase of %BF at 30ˈ (P<0.001). A similar increase is described by Dixon et al. 10 at 20ˈ POST, by Androustos et al. 15 at 30ˈ POST (after water or electrolyte drink), and also in other studies immediately after consumption 11,16. Furthermore, the growth of %BF throughout the measurement period was found by Dixon et al. 9 for 591 ml water or carbohydrate/electrolyte drink at times 20-60ˈ, by Androustos et al. 15 in consumption of 750 ml electrolyte drink at times 30-120ˈ and by Ugras 12 in consumption of 500 ml water each 15ˈ during one hour. Conversely, the study by Heiss et al. 17 even showed no statistically significant increase in %BF after consuming a 350 ml Gatorade drink, which may be due to the amount and type of drink used. However, the statistically significant difference between IST and CON occurred only 30ˈ POST (P<0.05), i.e., approximately at the time when the drink is still present in the stomach. The presence of drink in the stomach probably alters impedance values obtained from the body torso area. These affected/altered values are then used in the equations to calculate %BF, which may change the results and partially explain observed differences.

The amount of **VF** couldn´t be reliably determined even 90ˈ POST IST consumption. A statistically significant increase in values over CON occurred at the time of 30ˈ, 60ˈ, and 90ˈ POST (P<0.001). Our study is the first to focus on the effect of IST drinking on VF; thus, there are no previous studies to comment on. However, VF results were mainly affected by changes in BM and impedance in the body torso area, which was caused by the intake of fluids 14. The impedance value measured in the torso significantly affects the impedance of the whole body because this is the largest part of the body and the impedance values are lower than in other areas of the body. Fluids remain in the stomach for some time, distorting the results (as described above). So, a comparison of VF values with the impedance values measured in the torso at different time intervals would undoubtedly be more accurate in further research describing VF changes.

The analysis showed no statistically significant differences in **%BW** between IST and CON at any observed times. After consumption of the IST, there was first a statistically significant decrease (P<0.01) from BASELINE and then a statistically significant increase at 60ˈ (P<0.001) and 90ˈ (P<0.01) compared to 30ˈ POST. Similar results showed several studies after water consumption or hypotonic rehydration beverage 10,12,14 or isotonic drink 10,12,13. On the other hand, Dixon et al. 9 didn´t find any significant changes in total body water.

In our study, we have not shown the statistically significant growth of **I100** after IST consumption, no differences between IST and CON, and finally, no interaction between consumed fluid and time factor. The same results found Dixon et al. 9 after drinking a nearly similar amount of carbohydrate/electrolyte drink (591 ml), continued Matthews et al. 13 (466 ml of isotonic saline), Deurenberg et al. 19 (200 ml of tea) or Elsen et al. 20 1000 ml water and 700 ml of rehydration solution.

But if we focused on **I20**, it was significantly increased at 30ˈ POST over the baseline value (P<0.001) and at 60ˈand 90ˈ decreased under the value of 30ˈ (P<0.001), respectively (P<0.01). Also, the interaction between consumed fluid and time factor was statistically significant (P<0.001), and statistically significant differences (P<0.001) also appeared between IST and CON at 30ˈ. Dixon et al., in their another study 10, found a statistically significant increase in impedance 20ˈ after drinking nearly a similar amount of carbohydrate/electrolyte drink (591 ml), as we used. Although the effect of the quantity and composition of the beverage used is possible (similar or smaller amounts of drink were drunk but differed from one another in terms of sugar and sodium content), the effect of the type of BIA used on the impedance value in individual studies seems most likely. Dixon et al. used in the first study 9 leg-to-leg-BIA (50 kHz), as well as Matthews et al. 13, and arm-to-leg BIA (50 kHz) was used by Deurenberg et al. 19. Whereas in the second study, Dixon et al. 10 used segmental BIA, like us. In addition, the frequencies of 20 and 50 kHz are very similar, then 100 kHz and 20 kHz, which could have another connection with our impedance results. Other studies also describe statistically significant increases in impedance but in connection with the intake of much higher amounts of fluids 14,15.

But if we focus on changes in impedance in individual segments of the body, after observing the rest mode before the measurement, fluids will move from the upper limbs to the trunk, which leads to a decrease in impedance in the trunk 21,22, 23. After consuming liquids, the amount of water in the trunk increases even more, which corresponds to a significant decrease in the impedance of B100 and B20 below the baseline in all monitored times and, on the contrary, a gradual increase in the impedance of CON, which was statistically significant at a higher frequency of 100 kHz up to 60ˈand 90ˈ and at a lower frequency 20 kHz already in 30ˈ-90ˈ. This result is related to the standing position of the body during the measurement, while supine position would probably not have such a significant reduction in impedance in the trunk and the fluids would be more evenly distributed in the body 21. Due to the standing position during the measurement, despite the consumption of IST, there was a statistically significant increase in impedance at RA20 at 30ˈand 60ˈ and at LA20 at 90ˈ, while there was no statistically significant change for CON at RA20 to 90ˈand at LA20. When comparing changes in impedance over time for both RA100 and LA100, no statistically significant differences were demonstrated. It is a question whether it can be related to the fact, the higher frequency (100 kHz) resulted in less impedance than the lower frequency (20 kHz) 23. The comparison of impedance changes over time on the lower limbs did not yield any statistically significant results, which is probably related to the time that was not long enough for the changes after fluid consumption to be reflected in the lower limbs as well.

The resulting differences in impedances in segmental impedances and the position during the measurement show that the measurement in supine position should be methodologically more accurate. This assumption points to another avenue of possible research, where BIA would be used, which allows both supine and standing measurements with subsequent comparison of segmental impedance results with the same study design as ours. With a larger number of research participants and equal gender representation, it would also be beneficial to compare segmental impedances and BC parameters between women and men. Another enrichment of the study could be supplementing the consumption of tap water. It is necessary to compare impedance changes in individual segments when consuming tap water and IST, because water-electrolyte-balance is not maintained following acute hydration change, and the IST might have a greater effect on BIA than the simple tap water 24. Furthermore, from the detailed synthesis of the results of the above-described studies and our study, water appears not only to affect the increase in %BF in the time of 0-30ˈ POST but also in later time periods of 60-120ˈ POST. In contrast, isotonic drinks containing sugar and sodium affect %BF mainly at 0-30ˈ POST, and any prolonged temporal effects may be related to consumption of larger amounts of drinks (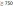 ml) 15.

## Conclusions

Incorrectly executed BC measurements can skew the results and interpretation of important BC indicators, which can affect the overall preventive or medical care of individuals in the public health system. Our study describes the changes in segmental impedances and selected BC parameters in healthy, young normal-weight adults after consuming 600 ml of IST at different time points and how it is possible to overestimate the monitored parameters. From the point of view of design, our research is significant mainly due to the choice of a time interval of up to 90ˈ time between the intake of IST and the analysis of segmental impedances and BC, a higher number of participants than has been included in similar studies so far and a detailed comparison of impedance values in different body segments, while maintaining the same technique measurement (BIA) and a similar amount of drink as used in similar studies.

Our research confirmed that BM is statistically significantly influenced by the amount of fluids consumed, as well as impedance in the trunk and upper limbs, which is also confirmed by the results of other parameters based on impedance values (VF and %BF). However, it should be taken into an account that even the position during the measurement can probably have a significant effect on the value of impedance, VF and %BF.

Therefore, based on our results we recommend the following. Our results of segmental impedances and selected BC parameters in healthy young normal-weight adults requires strict adherence to fluid restriction at least 90 min before the measurement to avoid false overestimation of BM, false impedance values, and the associated false overestimation of VF and %BF.

This information is essential for researchers, clinicians, and nutritionists who use SMF-BIA technology in public health care. However, in the case of food consumption, the recommendations may be different, and further analyses and comparisons with fluids consumption are needed.

## Figures and Tables

**Table 1:** Subjects' characteristics

	Women (n=53)		Men (n=42)		All (n=95)	
	Mean ± s.d.	Range	Mean ± s.d.	Range	Mean ± s.d.	Range
**Age (years)**	23.7 ± 1.5	20.0 - 29.0	24.1 ± 1.7	22.0 - 31.0	23.9 ± 1.6	20.0 - 31.0
**BM (kg)**	59.3 ± 6.5	46.7 - 73.9	79.7 ± 12.9	54.7 - 111.3	68.3 ± 14.1	46.7 - 111.3
**%BW**	54.8 ± 3.9	44.2 - 66.5	60.0 ± 4.6	47.4 - 68.8	57.1 ± 5.0	44.2 - 68.8
**%BF**	25.2 ± 5.4	8.9 - 39.5	18.0 ± 6.2	6.3 - 35.0	22.0 ± 6.8	6.3 - 39.5
**VF (cm2)**	43.7 ± 14.8	17.8 - 89.3	71.4 ± 28.6	33.2 - 151.0	55.9 ± 25.9	17.8 - 151.0
**I100 (Ω)**	663.7 ± 55.6	508.4 - 808.0	529.8 ± 58.7	385.9 - 704.0	604.5 ± 87.6	385.9 - 808
**I20 (Ω)**	738.8 ± 59.0	595.0 - 891.4	604.5 ± 63.5	447.0 - 799.8	679.4 ± 90.4	447.1 - 891.4
**B20 (Ω)**	27.2 ± 2.4	21.3 - 33.8	24.4 ± 2.6	17.4 - 33	25.9 ± 2.8	17.4 - 33.8
**B100 (Ω)**	23.6 ± 2.2	18.6 - 29.9	20.4 ± 2.4	14 - 27.8	22.2 ± 2.8	14 - 29.9
**LL20 (Ω)**	291 ± 26.3	221.8 - 357.4	260.7 ± 32.2	196.8 - 359.5	277.6 ± 32.7	196.8 - 359.5
**LL100 (Ω)**	260.7 ± 23.9	199.2 - 325.9	228.2 ± 29.1	169.3 - 318.7	246.3 ± 30.9	169.3 - 325.9
**RL20 (Ω)**	293.4 ± 27	230.1 - 365.8	260.6 ± 32.5	193.1 - 362.2	278.9 ± 33.7	193.1 - 365.8
**RL100 (Ω)**	263 ± 24.7	205.7 - 330.8	228.6 ± 29.3	167 - 322.1	247.8 ± 31.8	167 - 330.8
**LA20 (Ω)**	423.7 ± 36.4	334 - 540.2	321.9 ± 34.3	234.7 - 424.2	378.7 ± 61.8	234.7 - 540.2
**LA100 (Ω)**	382.6 ± 35.1	217.1 - 485.2	283.7 ± 32.5	173.6 - 377	338.9 ± 59.7	173.6 - 485.2
**RA20 (Ω)**	415.2 ± 37.2	338 - 522.1	316.9 ± 33.7	226.9 - 410.9	371.8 ± 60.5	226.9 - 522.1
**RA100 (Ω)**	373.7 ± 35.4	269.5 - 476.6	278.3 ± 32	174.8 - 361.5	331.5 ± 58.3	174.8 - 476.6
